# Seasonal dynamics of the wild rodent faecal virome

**DOI:** 10.1111/mec.16778

**Published:** 2022-11-11

**Authors:** Jayna Raghwani, Christina L. Faust, Sarah François, Dung Nguyen, Kirsty Marsh, Aura Raulo, Sarah C. Hill, Kris V. Parag, Peter Simmonds, Sarah C. L. Knowles, Oliver G. Pybus

**Affiliations:** 1 Department of Biology, University of Oxford; 2 Department of Pathobiology and Population Sciences, The Royal Veterinary College; 3 Institute of Biodiversity, Animal Health, and Comparative Medicine, University of Glasgow; 4 Nuffield Department of Medicine, University of Oxford; 5 School of Biosciences, University of Exeter; 6 University of Turku, Finland; 7 MRC Outbreak Centre, Imperial College London

## Abstract

Viral discovery studies in wild animals often rely on cross-sectional surveys at a single time point. As a result, our understanding of the temporal stability of wild animal viromes remains poorly resolved. While studies of single host-virus systems indicate that host and environmental factors influence seasonal virus transmission dynamics, comparable insights for whole viral communities in multiple hosts are lacking. Leveraging non-invasive faecal samples from a long-term wild rodent study, we characterised viral communities of three common European rodent species (*Apodemus sylvaticus, A. flavicollis,* and *M. glareolus)* living in temperate woodland over a single year. Our findings indicate that a substantial fraction of the rodent virome is seasonally transient and associated with vertebrate or bacteria hosts. Further analyses of one of the most common virus families, *Picornaviridae,* show pronounced temporal changes in viral richness and evenness, which were associated with concurrent and up to ~3-month lags in host density, ambient temperature, rainfall and humidity, suggesting complex feedbacks from the host and environmental factors on virus transmission and shedding in seasonal habitats. Overall, this study emphasizes the importance of understanding the seasonal dynamics of wild animal viromes in order to better predict and mitigate zoonotic risks.

## Introduction

Our knowledge of the global virosphere has rapidly expanded (C. X. Li et al., 2015; Roux et al., 2016; Shi et al., 2018; Zhang et al., 2018), mainly due to decreasing costs and increasing efficiency of high-throughput sequencing. However, while it is now relatively straightforward to genetically characterise host viromes and discover new virus sequences, most studies provide only a glimpse of the circulating virus diversity due to infrequent, non-systematic, and spatially limited sampling of target species. As a result, it is unclear why some viruses are found in some species or populations at specific time points but not in others (Harvey & Holmes, 2022).

While viral discovery studies provide valuable data for understanding the evolutionary history and host range of viruses, they offer limited insights into what factors shape wild animal viromes. In order to understand viral dynamics in wild populations, we need to move from descriptive host-virus associations to a mechanistic understanding of where and when viruses are transmitted and how entire viral communities (viromes) are shaped by the environment and local host communities (Bergner et al., 2019; Fearon & Tibbetts, 2021). For example, both decreases and increases in the number of parasites (i.e., richness) in wild animals have been associated with habitat loss and fragmentation (Mbora & McPeek, 2009; Morand et al., 2019), indicating that anthropogenic-mediated changes in host species composition and population densities can directly impact parasite community compositions. Furthermore, the influence of anthropogenic land-use change on virus community compositions has also been observed for a broad range of taxa, suggesting it is a key determinant of host viromes (Campbell et al., 2020; Hermanns et al., 2021; Myer & Johnston, 2019; Susi & Laine, 2021). These findings further highlight why studying community traits, such as parasite richness, is critical for understanding and forecasting zoonotic risk over time and space.

Current knowledge about what factors shape viral communities in animals comes from a small but growing number of studies. A comparison of viromes from three parasitic wasp species reared in laboratory conditions suggests that host phylogeny influences viral community structure (Leigh et al., 2018). However, it is unclear whether viromes in wild animals are also commonly predicted by host evolutionary history. Indeed, a study of multiple wild waterbird species sharing habitats found discordance between the host phylogeny and virome composition (Wille et al., 2019). This finding suggests that interspecific interactions and transmission among waterbirds might break down the host phylogenetic structuring of viral communities in wild settings. However, as investigations into virus community dynamics in multi-host systems are limited, it remains uncertain how viral communities vary across host and viral taxa or ecological contexts. Virome composition can differ within species due to demographic and environmental characteristics. For instance, a survey of 24 vampire bat colonies found that virus richness was positively associated with younger age structure, lower elevation, and increasing anthropogenic influence (Bergner et al., 2019). Studies on waterbirds also similarly found higher viral richness in younger age groups (Wille et al., 2021, Hill et al., 2022), suggesting age structure is a critical determinant of virus diversity in wild animals.

Although evidence suggests that environmental and host factors influence viral communities in wild animals, these surveys have predominantly been cross-sectional, with any given population sampled at a single time point. As a result, we have a sparse understanding of temporal dynamics in wild animal viromes. Fundamental questions such as how viral diversity varies over time and what proportion of viruses are only detected intermittently or at certain times of year in seasonal environments remain unaddressed. These dynamic environments affect wild animals through seasonally varying birth and death rates and timing of specific behaviours, including mating and other social interactions. As a result, many factors affecting viral transmission vary seasonally (Altizer et al., 2006), such as recruitment of susceptible individuals, population density, and contact rates. Furthermore, virus survival in the environment can fluctuate throughout the year and influence onward spread. For instance, the environmental persistence of avian influenza viruses is higher at colder temperatures (Brown et al., 2007)

Consequently, it is not surprising that zoonotic virus surveillance studies in reservoir populations have consistently observed seasonal variation in virus prevalence in several host species, such as rodents, bats, birds, and racoons (Amman et al., 2012; Fichet-Calvet et al., 2007; George et al., 2011; Hirsch et al., 2016; Páez et al., 2017). However, except for a handful of studies focusing on specific virus families (e.g., paramyxoviruses in bats and influenza viruses in mallard ducks (Latorre-Margalef et al., 2014; Wille et al., 2018)), investigations into temporal variation in virus diversity in wild animals are rare, leaving a significant gap in our knowledge about viral community dynamics in changeable environments. For example, we may expect increases in viral richness during an animal’s breeding season, driven by higher (primarily intra-specific) contact rates. Alternatively, viral community richness or composition may respond to seasonal changes in climate, for instance, if ambient conditions affect viral persistence in the external environment (Brown et al., 2007; Sobsey et al., 1988), which could impact viral richness and abundance.

Rodents are a significant zoonotic reservoir globally, and Europe has been identified as a hotspot for rodent reservoir diversity (Han et al., 2015). Furthermore, viral metagenomic surveys confirm that wild rodents carry a high and diverse viral burden, which includes several viruses closely related to human pathogens (Drexler et al., 2012, 2013; Firth et al., 2014; Kapoor et al., 2013; Phan et al., 2011; Williams et al., 2018; Wu et al., 2018), including coronaviruses(Wang et al., 2020). Therefore, understanding the composition and dynamics of rodent viromes is an important goal that can help shed light on when and where these host communities may pose the greatest risk of zoonotic spillover to humans. However, our understanding of virus diversity in rodents and what shapes variation in rodent viromes within and among sympatric species remains limited.

To address these questions, we leveraged a long-term capture-mark-recapture study of several sympatric rodent species in Wytham woods, Oxfordshire. Specifically, we characterised the viromes of three common resident species, *Apodemus sylvaticus* (wood mouse), *Apodemus flavicollis* (yellow-necked mouse), and *Myodes glareolus* (bank vole). These three species are ubiquitous across Europe, particularly in woodland habitats. They have fast-paced life histories, with females capable of producing multiple litters in her lifespan, which is typically less than one year. To characterise seasonal variation in viral communities, we generated metaviromic data from pooled faecal samples collected longitudinally from each species over a single year. By combining local microclimate and demographic data from the same period, we explored key factors that predict seasonal variation in the wild rodent virome.

## Methods

### Study population

Wild rodents were trapped and sampled over a one-year period (January 2017 to January 2018) in Wytham Woods (51° 46’N,1°20’W), a 385-ha mixed deciduous woodland near Oxford, UK. Three common rodent species are regularly caught at this site: two species of *Apodemus* mice (*Apodemus sylvaticus* and A*. flavicollis,* with *A. sylvaticus* more abundant) and the bank vole (*Myodes glareolus*). These are non-group-living, omnivorous woodland rodents with overlapping home ranges that show seasonal variation in reproduction, mortality, diet (Watts, 1968) and social interactions (Raulo et al., 2021). One night of trapping on a single c. 2.4ha trapping grid was carried out approximately fortnightly year-round. Small Sherman traps (baited with six peanuts, a slice of apple, and sterile cotton wool for bedding material) were set at dusk and collected at dawn the following day. Newly captured individuals were PIT-tagged for unique identification. Faecal samples were collected from the bedding material with sterilized tweezers and frozen at -80°C within 10 hours of trap collection. Traps that showed any sign of animal contact (traps that held captured animals and trigger failures where an animal has interfered with the trap but not been captured) were washed thoroughly with bleach between trapping sessions to prevent cross-contamination. All live-trapping work was conducted with institutional ethical approval and under Home Office licence PPL-I4C48848E.

### Sample selection and processing

We randomly selected 133 individual faecal samples (57 *A. sylvaticus,* 25 *A. flavicollis,* and 51 *M. glareolus*). Five sampling intervals were defined, which took into account the breeding cycle of the three rodent species. These were 1) Jan-Feb 2017, 2) Mar-Apr 2017, 3) May-Jul 2017, 4) Aug-Oct 2017, 5) Nov-Jan 2017/18. Faecal samples were pooled by species and sampling interval, using equal aliquots of 40mg faeces per individual per pool. For the last sampling interval, where there were fewer individuals of *A. flavicollis* and *M. glareolus* available (2 and 7, respectively), greater masses of faeces per individual (150mg and 70 mg, respectively) were used for pooling to ensure sufficient material for sequencing. Complete sample information for each pooled sample is outlined in Table S1.

The samples were processed as follows to enrich for RNA within encapsulated viruses: 1) Frozen archived faecal samples were first pooled, then suspended in DNA/RNA Shield Stabilization Buffer (Zymo), vortexed thoroughly, and the supernatant was filtered through a 0.45nm pore filter; 2) RNase treatment (RNase One) to remove non-encapsulated RNA from the sample; 3) RNA extraction using Zymo Quick Viral RNA and RNA Clean and Concentrator 5 kits; 4) DNA digestion following RNA extraction; 5) ribosomal depletion with Illumina Ribo-Zero Plus kit, which allows for ribosomal RNA removal in human, mouse, rat, and bacterial samples, during sequencing library preparation. The Oxford Genomics Centre carried out sequencing library preparation, which included cDNA synthesis, and sequencing on Illumina NovaSeq 6000 platform.

### Viral genomes reconstruction

A total of 355,917,017 pair-end reads of 150 base-pairs (bp) were obtained after sequencing. Illumina adaptors were removed, and reads were filtered for quality scores≥30 and read length >45bp) using cutadapt 1.18 (Martin, 2011). A total of 352,872,111 cleaned paired-end reads were *de novo* assembled into 435,021 contigs by MEGAHIT 1.2.8 with default parameters (D. Li et al., 2015). Viral contigs were identified by comparing the assembled contigs against the NCBI RefSeq viral database using DIAMOND 0.9.22 with an e-value cutoff of <10^-5^ (Buchfink et al., 2014). To eliminate false positives, all contigs that matched virus sequences were used as queries to perform reciprocal searches on NCBI non-redundant protein sequence database with an e-value cutoff of <10^-5^ (Altschul et al., 1990). We considered each viral contig as a viral operational taxonomic unit (vOTU). The abundance of each vOTU contig was assessed by iterative mapping reads against each contig using BOWTIE2 2.3.4.3 (Langmead, 2010) and BBMap 35.34 (Bushnell, 2014). For viral contigs corresponding to complete or nearly complete contigs, we examined Open Reading Frames (ORFs) using ORF finder (parameters: minimum ORF size of 300 bp, standard genetic code, and assuming there are start and stop codons outside sequences) in Geneious prime 2019.1.1 (Kearse et al., 2012) to exclude misassembled genomes.

Information on the number of raw, cleaned, and viral sequence reads per pooled sample are outlined in Table S2. Output data (blast results, viral contigs, read abundance) from the bioinformatic analyses can be found on DRYAD https://doi.org/10.5061/dryad.612jm645s

### Virus abundance and diversity metrics

After the assignment of contigs to vOTU, we normalised the abundance of contigs to the total reads and individuals used in a pool. To reduce the impact of contamination in our analyses, we excluded viral contigs with less than one read per 10 million. The abundance of viruses was then compared using normalised read abundance. Virus diversity was assessed using the number of virus genera (hereafter ‘richness’) and the evenness of virus genera (hereafter ‘evenness’), which was measured by calculating the Shannon entropy of virus genera in the community using the Shannon diversity index function in R library vegan. Consequently, viral evenness ranges from 0 and 1, and indicates the degree to which the virus community is dominated by a particular genus (i.e. evenness = 0) or whether different genera are equally abundant (i.e. evenness = 1). To identify unique and shared viruses across all time points, we visualised the distribution of viral contigs (200 bp or longer) with a minimum of 20 reads among host species with Venn diagrams (Yan, 2021). To determine if our methods were capturing the majority of virus genera in the system, we used rarefaction curves to assess the saturation of virus richness. We then calculated additive partitioning diversity to quantify how virus richness varied between species and time points (Oksanen et al., 2020). Finally, to assess how virus composition changes over time and which virus genera shift through time, we undertook a hierarchical PERMANOVA analysis (Anderson, 2001), with sampling intervals and host species as covariates and constraining permutations to within species only). Together, these analyses inform how sufficient these sampling efforts are for understanding wild animal viromes.

To reconstruct the picornavirus phylogeny, we assembled a multiple protein sequence alignment of 93 whole picornavirus genome sequences from the NCBI RefSeq viral database and eight picornavirus genome sequences identified in this study. Maximum-likelihood phylogeny was inferred with IQ-TREE v. 2.1.3 (Minh et al., 2020) using the best substitutional model identified by ModelFinder (Kalyaanamoorthy et al., 2017)

### Predictors of picornavirus richness and evenness

We evaluated drivers of two outcome variables – picornavirus richness and evenness – using Gaussian distributed generalised linear models (GLMs). We modelled picornaviruses in wood mice and bank voles separately and only modelled these virus-host combinations as up to 6 picornaviruses were found, and these hosts were sampled for viruses at each interval throughout the year. Four predictor variables with time series covering the preceding relevant seasons (June 2016 – Dec 2016) and picornavirus characterization period (Jan 2017-Dec 2017) were used to identify significant environmental and population factors affecting picornavirus richness and evenness. Temperature, humidity, and rain data were collected hourly at two microclimate stations within the woodlands. Host population density for each species was measured by the minimum number of known alive per hectare based on bimonthly trapping events across a 2.4ha grid between November 2016 and January 2018. Since predictor and outcome variables were calculated at different frequencies (daily to seasonally), we used locally estimated scatterplot smoothing (LOESS) and generalised additive models (GAMs (Wood, 2011)) to model a continuous estimate of each variable over the study period (June 2016 – Jan 2018 for predictor variables; Jan 2017-Jan 2018 for outcome variables). Bimonthly estimates for picornavirus richness, picornavirus evenness (see ‘Virus abundance and diversity metrics’ in Methods), and host population density were inferred with LOESS, while bi-monthly estimates for microclimate data (temperature, humidity, and rain) were inferred with GAMs.

Environmental and host density impacts may have delayed effects on observed picornavirus richness and evenness. Therefore, we first identified the appropriate time lags (if any) for each predictor variable. Significant relationships between picornavirus 1) richness and 2) evenness and the four predictors were identified for each diversity metric and host species using cross-correlation analysis. Cross-correlation analysis (ccf function in R) compares two time series and identifies similarities between the variables. Values range from -1.0 to 1.0; the closer the absolute value is to 1.0, the more linked the two variables are across time. In addition to identifying contemporaneous correlation, cross-correlation can be used to evaluate if there are lagged correlations (i.e. delayed but significant similarities between time series). We evaluated lags from 0 to 14 weeks in two weeks increments and identified significant residual auto-correlation values for each increment. If multiple lags were identified as significant for a given predictor variable, we selected the lag with the highest significant residual auto-correlation value (see Table S3) to use in GLM construction below. The maximum lag was set at 14 weeks to reflect the average lifespan of the wild rodents in the study (approximately three months).

We considered four separate GLMs per host species and diversity metric (i.e. AS vs viral evenness, MG vs viral evenness, AS vs viral richness, and MG vs viral richness) to evaluate drivers of picornavirus diversity. However, prior to undertaking a GLM analysis, correlations among the four variables (with or without lags as determined by the cross-correlation analysis; Table S9) for each metric and host species were visually assessed in each GLM in R using the library “corrplot” (Wei & Simko, 2021). If the correlation coefficient was 0.7 or greater (Figure S1), we reduced the sets of GLMs considered accordingly (Table S3). We used the library “AlCmodvg” (Mazerolle, 2020) for model selection, which considers the Akaike Information Criterion (AIC) and the number of parameters to determine the best fit model. Lastly, the GLM results were plotted using the library “jtools” (Long, 2020). Statistical analyses and most plots were undertaken in R version 4.1.1 (The R Core Team, 2021). Adobe Illustrator 2021 was also used to visualize the abundance of common vertebrate-associated and bacteriophage viruses over time.

## Results

### Virome dynamics in wild rodents

Over a one-year period (January 2017 to January 2018), we characterised viruses in faeces from a total of 133 individual rodents (57 *A. sylvaticus,* 25 *A. flavicollis,* 51 *M. glareolus*). For each of the five 2-3 month sampling intervals, we randomly selected up to 13 individual samples per species to create species and sampling interval-specific pools for metagenomic sequencing (see Methods, Table S1 for further details). This approach resulted in five pools for both *A. sylvaticus* (wood mouse) and *M. glareolus* (bank vole) and three pools for *A. flavicollis* (yellow-necked mouse) which are less abundant at the sampling site.

**Figure 1:**
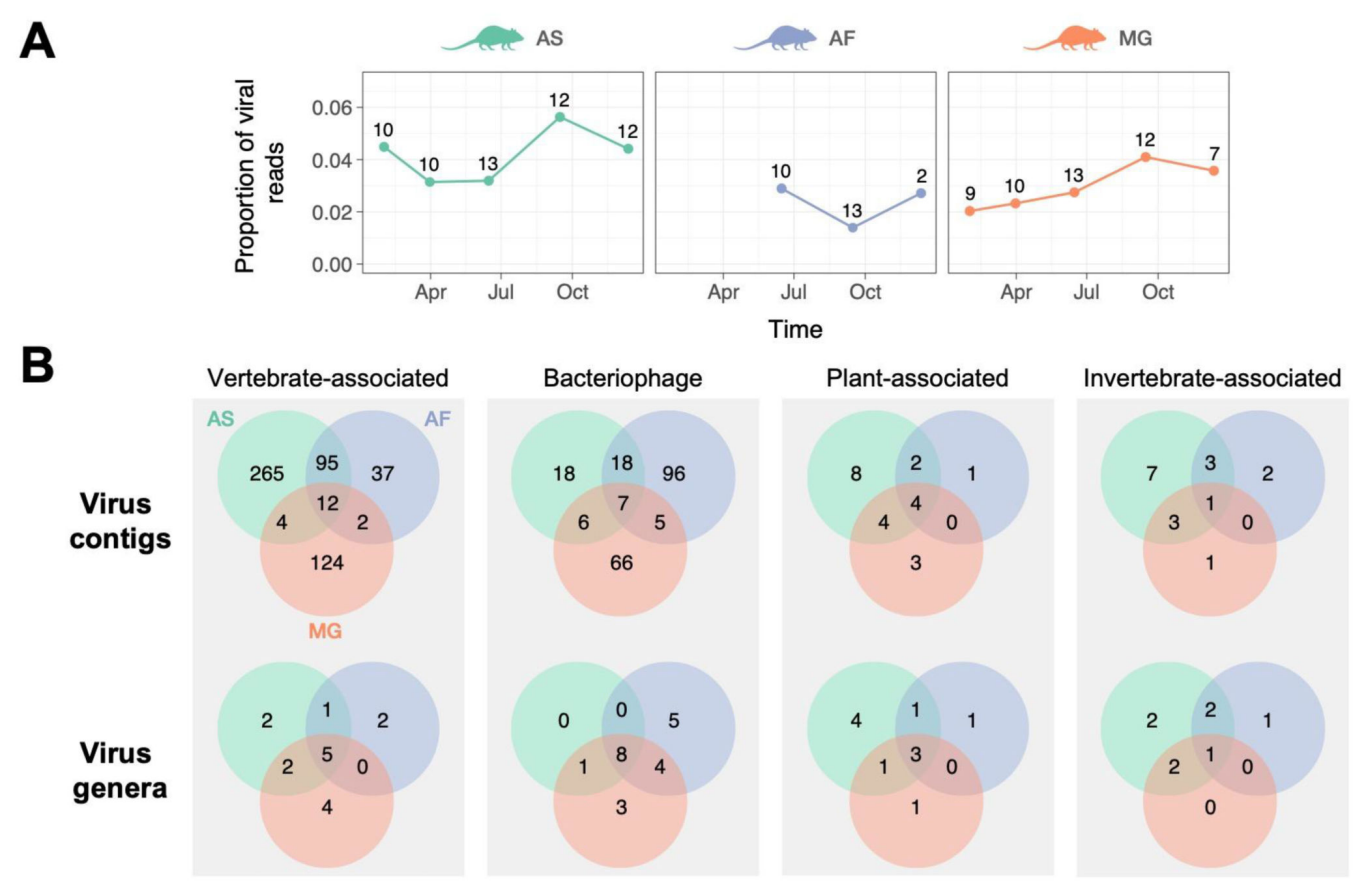
Summary of viral reads detected in the wild rodent faecal virome in Wytham woods over a single year. A) Proportion of viral reads detected through time for the three host species (AS = wood mouse, AF = yellow-neck mouse, and MG = bank vole). Proportion is based on the number of reads per sequencing library i.e. read depth. Numbers above each point indicate the number of individuals included in each pooled sample. Timepoints correspond to the midpoint of the sampling interval (see main text). B) Distribution of viral contigs and genera across four main host groups (based on minimum contig size of 200 nucleotides, applying a minimum threshold abundance of 1 read per 10 million in each pooled sample, and restricting to contigs with at least 20 reads). Green, blue, and orange corresponds to AS, AF, and MG, respectively.

Of the total quality-filtered and trimmed reads, 3.20% (~22.7M/711.8M) were taxonomically assigned to known viruses (see Methods). Figure 1 provides an overview of the viruses detected across all rodent species (hereafter, ‘Wytham rodents’). Clean virus abundance ranged from 1.06M to 2.88M reads per pooled sample (Table S2), with median abundances of 2.27M, 1.40M, and 1.22M for wood mice, yellow-necked mice and bank voles, respectively, with the proportion of viral reads (number of viral reads / total number of sequenced reads) varying somewhat among species and throughout the year (Figure 1A). Although the number of individuals per pooled sample varied between 2-13, this was not significantly correlated with the number of virus genera (i.e., viral richness) in each pooled sample (Pearson correlation = 0.2596; p = 0.39). Rarefaction curves further suggest that viral richness is approaching saturation in the Wytham rodents (Figure S2), indicating that additional sampling is unlikely to reveal many more viral genera.

The majority of virus contigs are associated with virus families that infect vertebrates or bacteria (Figure 1B). This observation is somewhat unexpected as the viral enrichment protocol used in this study was optimised for characterising viral RNA in encapsulated viruses (see Methods), regardless of their host association. Specifically, the number of bacteriophage contigs is notable (Figure 1B) since most bacteriophages have double- or single-stranded DNA genomes, although our protocols should also detect DNA viruses undergoing active replication or transcription. Alternatively, bacteriophages could be preferentially enriched in shotgun metagenomic datasets due to their large genome sizes (>100kb) (Dion et al., 2020). While this might be a contributing factor, the most abundant bacteriophage virus families in the Wytham rodent virome were *Leviviridae* (+ssRNA) and *Microviridae* (ssDNA), which have genome sizes ranging from 4 to 6.5kb (Table S4).

While a substantial proportion of contigs were host species-specific (wood mice = 265/852 (31.1%), yellow-necked mice = 144/852 (16.9%), bank voles = 223/852 (26.2%), most vertebrate-associated viral contigs were detected in at least two host species (Figure 1B). Furthermore, a larger proportion of viral contigs (18.1%; 154/852) were shared between the two closely related mouse species (wood mouse and yellow-necked mouse) than between mice and voles (7.2%; 107/852).

**Figure 2:**
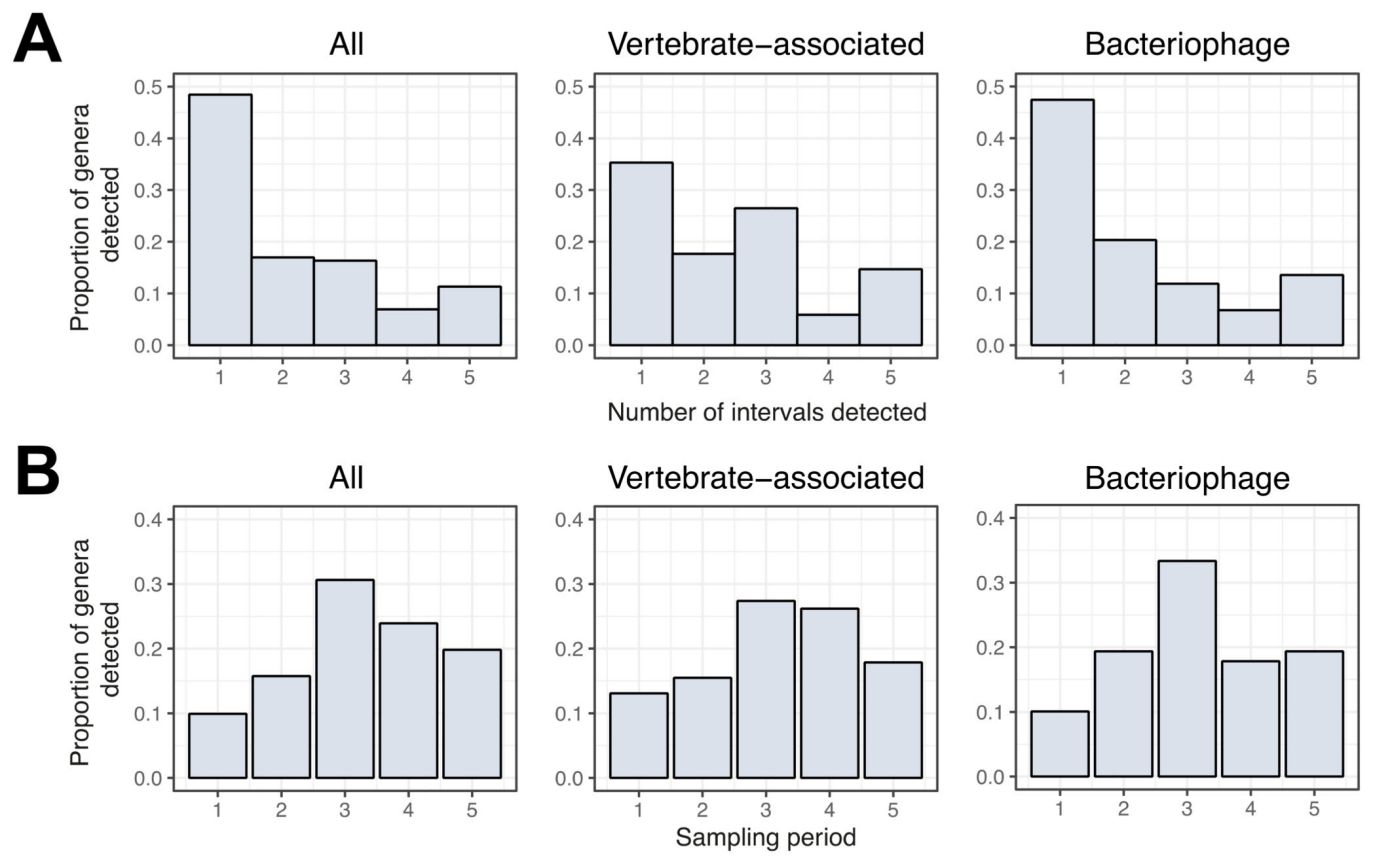
Variation in virus detection across the year. A) Histogram showing the proportion of viral genera by the number of times they were detected across the five sampling intervals for all viruses, vertebrate-associated viruses, and bacteriophages. B) Histogram summarising the proportion of viral genera detected in each sampling period (1 = Jan-Feb 2017, 2 = Mar-Apr 2017, 3 = May-Jul 2017, 4) Aug-Oct 2017, 5) Nov-Jan 2017/18) for all viruses, vertebrate-associated viruses, and bacteriophage.

To understand how virus detection varies across the year, we quantified the proportion of viral genera detected across all three hosts by the number of times it was detected (Figure 2A) and in each sampling interval (Figure 2B). Overall, 65.4% (104/159) of viruses were only observed in one or two intervals. A similar trend was noted for both vertebrate-associated viruses (18/34 = 52.9%) and bacteriophage (40/59 = 67.8%), indicating that most viruses in wild rodents are observed intermittently. The proportion of detected viruses varied seasonally, with the highest percentage observed in the third sampling period (Figure 2B), which corresponds to spring/summer months when host population density is low (Figure S3). Furthermore, most viruses (255/343 = 74.3%) were detected between the third and last sampling periods, i.e., the spring/summer and autumn/winter months (Figure 2B).

The relative abundance of viruses (Figure 2) is affected by changes in both virus occurrence between individuals and abundance within individuals. Sample pooling does not allow us to disentangle these; therefore, it is likely that viruses at low prevalence across the population or low virus abundance within individuals are not detected. However, we can still determine how much variation in viral richness (genera level) was observed at different levels – within a (pooled) sample, between species, and between sampling periods (Table 1) - with additive diversity partitioning (Crist et al., 2003). When considering viromes of all three host species together, around a third of virus richness (28.5%) was observed within pooled samples, 18.5% was observed between pooled samples within a given sampling period (i.e. among species, Table 1), while over half of all virus richness (53%) arose between sampling periods. In both wood mice and bank voles, approximately equal proportions of viral richness occurred within samples (45-47%) and between sampling periods (53-55%). However, in yellow-necked mice, the proportion of virus richness across sampling periods was lower (42.6%) than in the other host species (Table 1). This difference likely reflects sampling bias, particularly as faecal samples from yellow-necked mice were only available for three of the five sampling periods. Nevertheless, these findings suggest a significant change in virus richness through time in Wytham rodents and that the structure of viral communities is highly transient.

To understand how the viral community composition differs between host species and sampling intervals, we undertook a hierarchical PERMANOVA analysis (Anderson, 2001). Overall, we found that the virus community composition significantly shifts over time (p < 0.007), with host species having a weaker effect (p < 0.043) (Table S5). However, sampling interval and host species were not significant when considering vertebrate-associated or bacteriophage viruses separately (Table S5). We also identified the main virus genera that shift between sampling intervals (Figure S4), which included *Eucampyvirinae* (family: *Myoviridae*bacteriophage), *unclassified Picobirnaviridae* (family: *Picobirnaviridae* – vertebrate-associated), *Mamastrovirus* (family: *Astroviridae* - vertebrate-associated)*, Cardiovirus* (family: *Picornavirus* - vertebrate-associated), *unclassified Dicistroviridae* (family: *Dicistroviridae* – invertebrate-associated).

### Extensive circulating virus diversity

**Figure 3:**
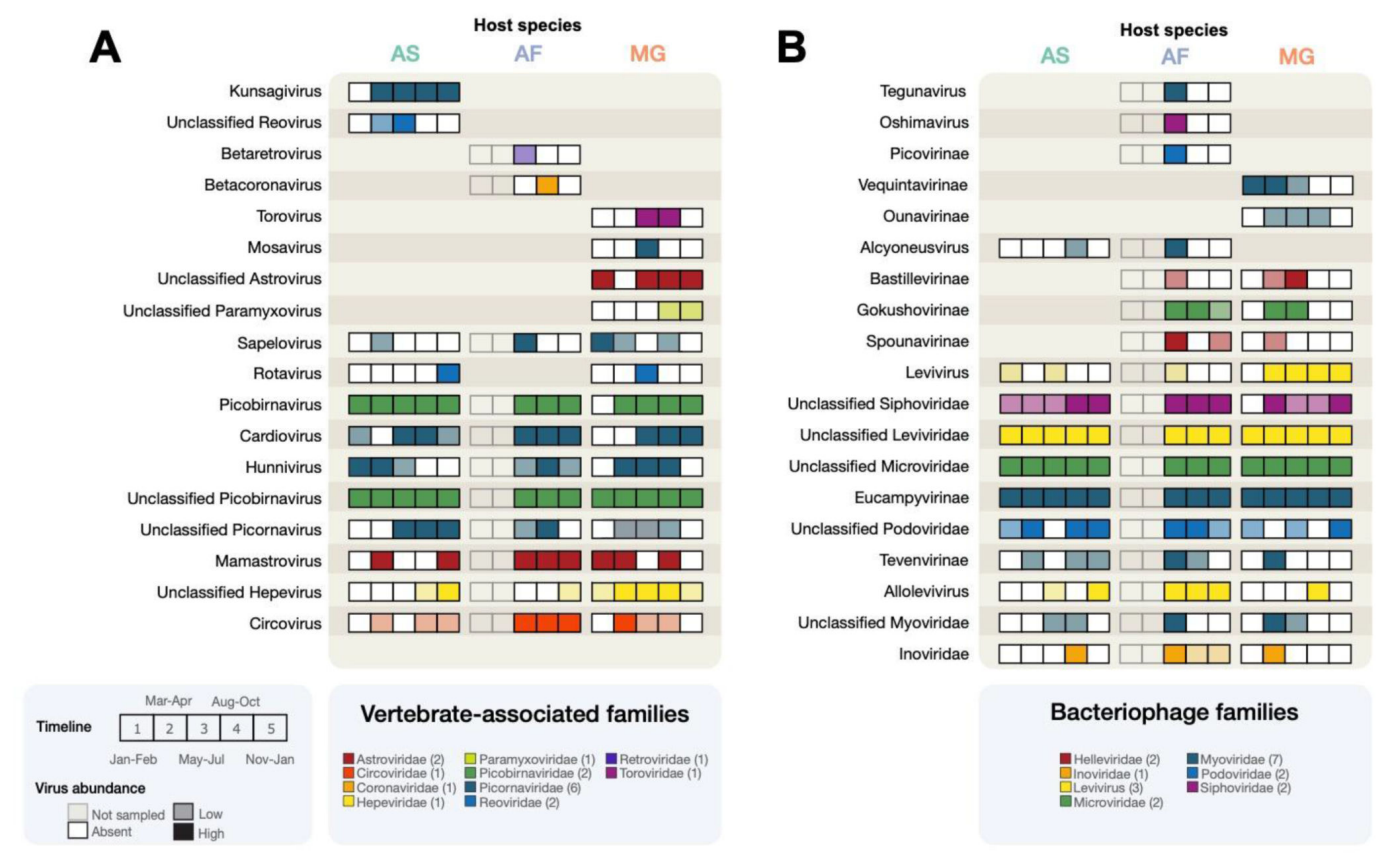
Temporal patterns of vertebrate-associated and bacteriophage virus genera in Wytham rodents. A) Vertebrate-associated virus genera. B) Bacteriophage virus genera Each box within a set of five corresponds to a distinct sampling interval (AF was only sampled at three intervals). White boxes indicated tested timepoints without the virus genus but that it was present in that host species at other timepoints. Shaded boxes indicate the presence and are coloured by virus family - solid-filled boxes indicate at least 20 reads (high abundance), while light-shaded boxes indicate less than 20 reads (low abundance).

Closer examination of the temporal patterns of the vertebrate-associated and bacteriophage viruses confirmed that considerable virus diversity was detected in the Wytham rodents, corresponding to different virus families, genera, and genome architectures (i.e., single- or double-stranded, DNA or RNA genomes) displaying highly variable patterns of seasonal detection (Figure 3, Table S6). A solid-filled box indicates that at least 20 reads were detected for a virus genus in a particular host, a lighter-shaded box indicates less than 20 reads were detected, and white boxes indicate no reads were detected. The most abundant viruses belong to the virus families *Picobirnaviridae* (vertebrate-associated) and *Leviviridae* (bacteriophage), which were detected throughout the year at high read abundance (ranging from 0.66M-2.32M for *Picobirnaviridae* and 0.29M-1.35M for *Leviviridae* in pooled samples). Other common vertebrate-associated viruses were members of several non-enveloped ssRNA virus families, such as *Picornaviridae, Astroviridae, Hepeviridae,* and the dsRNA virus family, *Reoviridae.* We also detected multiple enveloped RNA and reverse-transcribing viruses (*Betaretrovirus*, *Betacoronavirus*, an unclassified *Paramyxovirus*, and a *Torovirus*) in yellow-necked mice and bank voles.

Apart from picornaviruses, a more resolved taxonomic classification of the most common vertebrate-associated and bacteriophage virus families, e.g., *Picobirnaviridae, Leviviridae,* and *Microviridae*, was not possible due to poor representation of these taxa in reference databases. As a result, it is challenging to ascertain more detailed information about these viruses. For example, which hosts does the bacteriophage infect and how many distinct virus species (i.e., virus genomes) are present? We aimed to partly address the latter by considering virus contigs similar in length to complete genomes (see Table S7), many of which are likely to represent new viruses. Based on this simple approach, there appear to be potentially 114 putative *Picobirnavirus* genomes (which are bisegmented), 21 putative *Levivirus* genomes, and nine putative *Microvirus* genomes (Table S7).

### Seasonal co-circulation of picornaviruses

**Figure 4:**
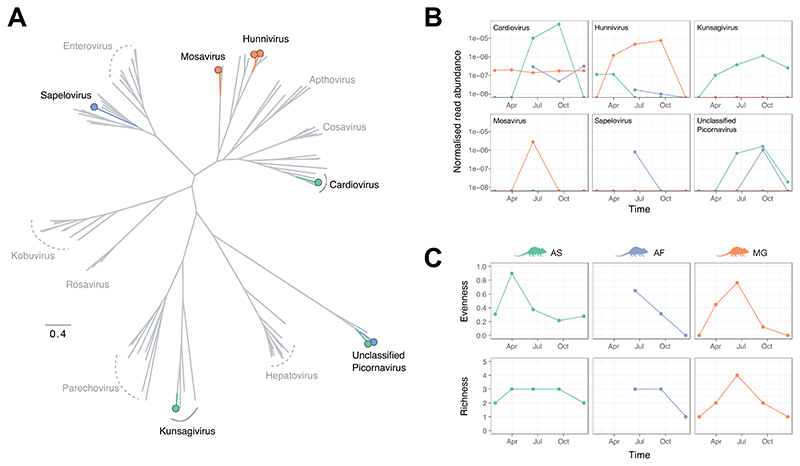
Picornavirus diversity and abundance. A) Evolutionary relationship of picornavirus assembled genomes identified in Wytham rodent faecal virome (coloured by their predominant host association) and a subset of known mammalian picornaviruses (in grey). B) Overall normalised read abundance of six picornavirus genera over time. Read counts per pooled sample below 1 per 10 million read threshold were excluded. Colours indicate association with host species (green, orange, and blue correspond to AS, MG, and AF). C) Diversity of picornaviruses, measured as virus evenness and richness, over time.

In Wytham rodents, picornaviruses were the most common and taxonomically well-characterised viruses. Furthermore, as they contain several important pathogens that affect human and animal health (e.g., *Enterovirus* and *Apthovirus),* we undertook a more detailed analysis to understand seasonal variation in picornavirus abundance and diversity. We assembled eight virus contigs for the most prevalent picornaviruses (see Table S8 for further details), representing partial and near-complete genomes. The eight genome sequences correspond to six distinct genera (Figure 4A) and share between 48 and 95% amino acid sequence identity with their closest BLAST hits, which were primarily associated with mammalian hosts, such as bats and other rodent species. The normalised read abundance (the number of viral reads divided by the total number of reads (i.e., read depth) per pooled sample and the number of individuals included in the pooled sample) showed strong seasonal variation for all six viruses (Figure 4B). Furthermore, the seasonal patterns of occurrence and peak abundance varied strikingly across these picornaviruses (Figure 4B). For example, *Mosavirus* and *Sapelovirus* were only observed at a single time point and most abundant in early summer (between May and July), while others (e.g., *Hunnivirus* and *Kunsagivirus)* were detected in multiple consecutive periods and reached peak abundance in late summer (between August and October). Importantly, this suggests that even for related viruses, there may be marked variation in the underlying drivers of transmission. Our data also demonstrated that while multiple distinct picornaviruses co-circulate in all three rodent species, for the most part, these viruses are disproportionately associated with a single host species (Figure 4B). In particular, the two distinct genome sequences of the “Unclassified Picornavirus” genus (Figure 4A) were exclusively found in wood mice or yellow-necked mice, but not both, even when these genomes were detected at the same sampling interval (Figure 4B). However, as the yellow-necked mice are less abundant than the other two species, sampling bias and the pooling of samples are likely to affect the observed virus sharing among host species.

Next, we investigated temporal patterns of picornavirus diversity (Figure 4C). Similar to virus abundance, viral evenness and richness exhibited seasonal variation. The trends are broadly consistent among the three rodent species, with the highest picornavirus diversity (evenness and richness) occurring between May and July in early summer. However, viral evenness peaked earlier in wood mice between March and April during the spring months. The pattern is likely driven by the presence of multiple picornaviruses that are at similarly low abundance in the wood mice, resulting in high viral evenness. As the most abundant picornavirus genus, *Cardiovirus,* becomes predominant among the picornaviruses at later time points, it leads to a concurrent decrease in viral evenness as the relative frequencies among co-circulating picornaviruses become unequal. A similar observation is observed in bank voles, where a notable reduction in viral evenness in late summer (August to October) coincides with a peak in *Hunnivirus* abundance.

When restricting the analysis to the eight picornavirus genomes, we observed broadly similar seasonally varying patterns in abundance, richness, and evenness at the species level (Figure S5). However, there were notable differences compared with the analysis undertaken at the genera level. In wood mice, a comparable peak in viral species evenness was absent, and a peak in viral species richness occurred in late summer/early autumn (Figure S5). As the eight genome sequences represent only 11% of contigs included in the genera-level analysis, it is difficult to determine if these patterns in virus species diversity are an accurate reflection of picornavirus dynamics or biased by known viral genomes.

### Drivers of picornavirus diversity

To explore the predictors of picornavirus diversity in Wytham woods, we focused on wood mice and bank voles, which were sampled in each of the five intervals. We evaluated three environmental variables (temperature, humidity, and rain) using data collected from June 2016 to January 2017 from two weather stations located within the woodlands, together with approximately fortnightly estimates of host population density, calculated as the minimum number known alive (MNKA) per hectare from trapping data. Time series data on picornavirus diversity and the four variables was reconciled using interpolation techniques (see Methods). Specifically, we used a fortnightly interval to derive estimates of all variables at the resolution available for the host density data (Figures S3 and S6). We undertook a cross-correlation analysis to select the single most informative time lag for each of the four variables (temperature, humidity, rain, and host population density), as identified by the highest correlation coefficient (Table S9). The maximum time lag was set as 14 weeks to reflect the expected average lifespan of wood mice and bank voles (~3 months). We found a notable correlation between picornavirus diversity and the ecological conditions experienced by host species in the preceding weeks and months (r_xy_ = 0.20-0.88; Table S9). For viral evenness, time lags in the four variables ranged from 10 to 14 weeks in wood mice and 6 to 14 weeks in bank voles, while for viral richness, time lags ranged from 2 to 14 weeks for both species (Table S9).

**Figure 5:**
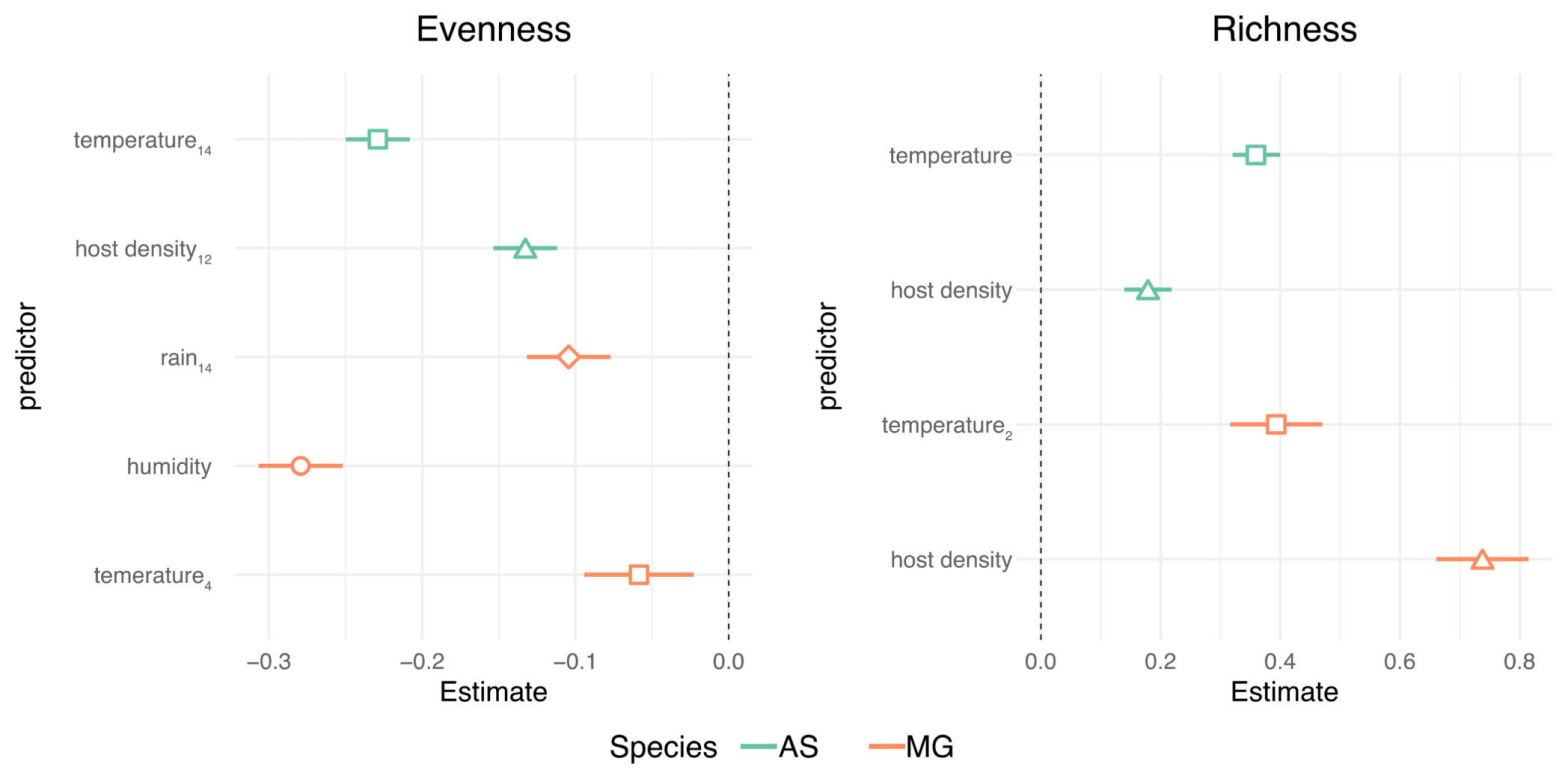
Predictors of picornavirus diversity. Standardised coefficients from the best fit models (mean-centred and scaled by one standard deviation) are illustrated for each diversity metric and species. Subscripts in variable names indicate time lag in weeks. AS = wood mice, MG = bank voles.

We constructed generalised linear models (GLMs) containing each time-lagged variable as predictors for each host species and diversity metric. Sets of GLMs were reduced accordingly to exclude highly correlated variables (i.e., >0.7; see Table S3, Figure S1). The results indicated that drivers of picornavirus diversity varied by species and diversity metric (Figure 5). For both species, the temperature in the preceding 1-3 months was negatively correlated with viral evenness (Figure 5, Table S10). In wood mice, viral evenness was additionally associated with a lower host population density three months previously, suggesting that the peak in viral evenness followed a period of low population density (in late winter) when the population mainly comprises overwintering individuals and when home ranges are largest and overlapping. In bank voles, viral evenness was negatively associated with rainfall in the previous three months and concomitant humidity (Figure 5, Table S10). Viral richness was positively associated with concomitant host population density and temperature in both wood mice and bank voles (Figure 5, Table S10)

Although we found similar predictors associated with viral evenness and richness in both host species, as the same set of predictors was not evaluated in each GLM, interspecific differences should be interpreted with caution. Specifically, the absence of a predictor in our analyses does not necessarily mean it is not associated with viral diversity. Therefore, to better understand the extent of interspecific variation in shaping virus diversity, additional field data will be required to characterise viral communities on finer temporal scales (e.g., fortnightly or monthly).

## Discussion

We examined the seasonal dynamics of the faecal virome in three wild rodent species widespread in the UK and Europe. Strikingly, we found extensive virus diversity circulating in these rodents throughout the year. Detected viruses were predominantly associated with vertebrate or bacteria hosts and represented a broad range of virus genomic organisation (RNA and DNA, single- and double-stranded), and virome diversity and community composition varied markedly throughout the year. Although viruses appear to be largely host-specific at the inferred species level, we saw substantial virus sharing among species, particularly among the wood mice and yellow-necked mice, indicating host phylogenetic relatedness is an important determinant of virus ecology. Furthermore, temporal patterns in virus abundance suggest marked variation in the epidemiology of co-circulating viruses, which can differ within and between species. Lastly, seasonal trends in picornavirus diversity suggest that these viral communities are shaped by biological and ecological processes, which likely influence within-host viral dynamics, environmental persistence, and between-host viral transmission.

Our study corroborates previous findings that rodents harbour a substantial and diverse virus burden in the gastrointestinal tract (Firth et al., 2014; Phan et al., 2011; Williams et al., 2018; Wu et al., 2018) with individuals likely encountering a shifting array of seasonally abundant viruses over their lifetimes (Abolins et al., 2018; Firth et al., 2014), contributing to their highly activated immune state (Abolins et al., 2017). Most vertebrate-associated virus genera identified in the Wytham rodents have been detected previously in wild rodents in the USA and China (Firth et al., 2014; Phan et al., 2011; Williams et al., 2018; Wu et al., 2018). *Cardiovirus* and *Picobirnavirus* have previously been reported in four major untargeted viral metagenomic surveys of wild rodents (Firth et al., 2014; Phan et al., 2011; Williams et al., 2018; Wu et al., 2018), suggesting these viruses are widespread and endemic in rodents. Two virus genera detected here have not previously been reported from wild rodents - *Kunsagivirus* (family *Picornaviridae)* and *Torovirus* (family *Tobaniviridae*). Although information about Kunsagivirus is limited (currently, only six sequences are available in Genbank), *Torovirus* is an enveloped virus commonly found in mammals, including humans, with gastroenteritis (Horzinek et al., 1987; Jamieson et al., 1998). Furthermore, the lower abundance of enveloped viruses than their non-enveloped counterparts is not surprising given their increased lability in the gastrointestinal tract. Although detailed characterisation of enveloped viruses in Wytham rodents was limited, contigs of *Paramyxovirus* detected in bank voles in this study closely matched another *Paramyxovirus* (genus *Jeilongvirus*) isolated from bank voles in Slovenia (Vanmechelen et al., 2018).

There was notable variation in observing a specific virus genus in the Wytham rodents across the year. Some viral genera from the most abundant virus families (*Picobirnaviridae* (vertebrate-associated), *Leviviridae* (bacteriophage) and *Microviridae* (bacteriophage)) were observed at all sampling intervals at high levels in all three species, indicating that they (or their bacterial hosts) persist in the population by establishing a chronic infection or environmental persistence which facilitates frequent reinfection. However, a significant fraction of virus diversity (104/159 genera) was detected only in one or two of five seasonal sampling periods. Hierarchical analysis of virus richness suggests there is substantial turnover in viral diversity in the Wytham rodents, with around half of diversity absent from each sample interval. Although these results suggest that wild rodents may support different virus epidemiological dynamics within a single year, more in-depth investigations will be required to understand the impact of pooling and sampling effort, particularly for viruses with low prevalence or abundance within individuals, which might appear transient despite continuous circulation. Importantly, however, these findings also highlight that crosssectional surveys will miss a large proportion of circulating virus diversity, even when samples are taken during times of the year when virus diversity is maximal, such as the spring and summer months in this population.

Despite sharing the same seasonal environment, the factors predicting picornavirus diversity differed between wood mice and bank voles. However, these interspecific differences should be interpreted carefully as different combinations of predictors were evaluated for each diversity metric and host species. Concomitant host population density and temperature predicted higher virus richness in both host species, suggesting higher intraspecific interactions and warmer conditions increase picornavirus transmission (and/or environmental persistence) and lead to a higher number of viral species circulating in the following months. Host density in the previous 2-3 months was associated with lower viral evenness in wood mice – several mechanisms could explain this pattern. For example, a higher population density could facilitate certain viruses to dominate transmission events through smaller home range sizes and reduced frequency of contacts, or the increase in density could affect competition and alter within-host replication dynamics. Future studies that incorporate more samples collected at higher frequencies could be used to test such hypotheses explicitly.

The widespread distribution of wood mice and bank voles in the UK makes them highly amenable for long-term field studies and have been previously leveraged to understand natural drivers of virus transmission in wildlife populations (Begon et al., 2009; Carslake et al., 2006; Knowles et al., 2012; Telfer et al., 2002, 2007). While these studies have focused on specific DNA viruses that are endemic in these species, they also observed heterogeneity in rodent virus epidemiology, including between years, host species, individuals, and across different viruses (Begon et al., 2009; Carslake et al., 2006; Knowles et al., 2012; Telfer et al., 2002, 2007).

We detected long time lags (~3 months) between some environmental variables and picornavirus diversity, particularly for viral evenness. This observation could be because pooled samples were from a time window, where individual samples from 2 to 3 months were aggregated into one ‘timepoint’. Although such temporal pooling is not ideal for time series evaluation, it provides a valid first approximation of important seasonal correlates of viromes and an improvement on previous cross-sectional surveys. We expect many viruses to be transmitted between conspecifics through close contact and between species via the environment. However, the ability of viruses to remain transmissible in the environment is highly variable across taxa. For example, hepatitis A virus (genus *Hepatovirus,* family *Picornavidae*) is very stable under a broad range of temperature, humidity, and pH conditions and can survive over three months in the environment (Sobsey et al., 1988). In contrast, other picornaviruses, such as Foot and Mouth disease virus (genus *Aphthovirus),* appear to be less stable in the environment, with longer survival times observed at higher humidity and moderate temperatures (Abad et al., 1994; Mbithi et al., 1991; Mielke & Garabed, 2020). Although we observed clear seasonality in picornavirus detection and abundance, given the substantial temporal turnover in viral diversity, it is reasonable to assume that other viruses in Wytham rodents also circulated seasonally, especially those detected transiently in the population (e.g., *Coronavirus, Paramyxovirus*). In the future, we plan to develop mechanistic transmission models in these systems using field studies with a higher temporal resolution. Mechanistic models could be adapted to other rodent systems to forecast peaks and troughs in epizootics and test potential interventions in settings where zoonotic viruses are a risk to human populations.

Understanding viral community dynamics is key to predicting and mitigating human risk from known and unknown rodent zoonoses. Improvements in sequencing technology that enable the identification and monitoring of RNA viruses longitudinally in wildlife are crucial to establishing the spatial, temporal, and environmental factors that determine zoonotic risk. Previous work has shown that specific rodent-borne zoonotic viruses exhibit strong seasonal dynamics in the reservoir population (Fichet-Calvet et al., 2007; Luis et al., 2015; Tian et al., 2017). Nevertheless, by quantifying the virome dynamics, we can identify the co-occurrence of a community of viruses, their transmission across the year, and associations with the environment and host ecology. This step moves our current knowledge about the seasonal dynamics of viral communities and contributes to a more comprehensive understanding of virus transmission ecology in wildlife populations.

## Supplementary Material

Supplementary Information

## Figures and Tables

**Table 1:** Hierarchical partitioning of total virus richness.

Host group	Mean virus richness (genera)	Level	%
All	25.1	Within sample	28.5
	16.3	Between species	18.5
	46.6	Between sampling periods	53.0
	88	Total	100
*A. sylvaticus*	21.6	Within sample	47.0
	24.4	Between sampling periods	53.0
	46	Total	100
*A. flavicollis*	28.7	Within sample	57.4
	21.3	Between sampling periods	42.6
	50	Total	100
*M. glareolus*	26.4	Within sample	45.5
	31.6	Between sampling periods	54.5
	58	Total	100

## Data Availability

The raw sequencing data generated in this study have been deposited in the Sequence Read Archive (BioProject ID: PRJNA803204) under the accession numbers: SRX14033113-SRX14033125. Assembled picornavirus genomes have been deposited in Genbank under the accession numbers: ON136174-ON136181. Data from the bioinformatics pipeline and metadata associated with this research is available on Dryad doi:10.5061/dryad.612jm645s, while associated code is available via Github: https://github.com/jnarag/Wytham-rodent-virome.

## Benefit-Sharing Statement

Benefits generated: Benefits from this research accrue from the sharing of our data and results on public databases as described above.
