# Supplementary material for: Seasonal dynamics of the wild rodent faecal virome
Source: Mol Ecol. 2022 Nov 23;32(17):4763–76. doi: 10.1111/mec.16778 (PMC7614976; doi:10.1111/mec.16778)
Supplement: Supplementary file 1 — Appendix S1. [file MEC-32-4763-s001.docx]

**Supplemental Information for:**

**Seasonal dynamics of the wild rodent faecal virome**

**Jayna Raghwani, Christina L. Faust, Sarah François, Dung Nguyen, Kirsty Marsh, Aura Raulo, Sarah C. Hill, Kris V. Parag, Peter Simmonds, Sarah C. L. Knowles, Oliver G. Pybus**

**Table of Contents:**

| **Table S1** | Page 2 |
| --- | --- |
| **Table S2** | Page 2 |
| **Figure S1** | Page 3 |
| **Table S3** | Page 4 |
| **Table S4** | Page 5 |
| **Figure S2** | Page 6 |
| **Figure S3** | Page 6 |
| **Table S5** | Page 7 |
| **Figure S4** | Page 7 |
| **Table S6** | Pages 8-11 |
| **Table S7** | Page 12 |
| **Table S8** | Page 12 |
| **Figure S5** | Page 13 |
| **Table S9** | Page 14 |
| **Table S10** | Page 15 |

***Table S1:*** *Sample information of each pooled sample*

| **Pool No.** | **Species** | **Sampling interval** | **No. of individuals per pool** |
| --- | --- | --- | --- |
| 1 | AF | 3 | 10 |
| 2 | AF | 4 | 13 |
| 3 | AF | 5 | 2 |
| 4 | AS | 1 | 10 |
| 5 | AS | 2 | 10 |
| 6 | AS | 3 | 13 |
| 7 | AS | 4 | 12 |
| 8 | AS | 5 | 12 |
| 9 | MG | 1 | 9 |
| 10 | MG | 2 | 10 |
| 11 | MG | 3 | 13 |
| 12 | MG | 4 | 12 |
| 13 | MG | 5 | 7 |
| **Total** | | | **133** |

***Table S2:*** *Raw, clean, viral read abundance, and number of viral genera observed for each pooled sample*

| **Pool No.** | **Species** | **Sampling interval** | **No. of raw paired-end reads** | **No. of cleaned paired-end reads** | **No. of viral reads** | **No. of viral genera** |
| --- | --- | --- | --- | --- | --- | --- |
| 1 | AF | 3 | 24,280,792 | 24,062,146 | 1,404,794 | 48 |
| 2 | AF | 4 | 25,640,698 | 25,409,164 | 1,194,326 | 39 |
| 3 | AF | 5 | 30,392,154 | 30,096,637 | 1,649,690 | 40 |
| 4 | AS | 1 | 26,239,174 | 25,992,596 | 2,355,360 | 17 |
| 5 | AS | 2 | 28,065,334 | 27,824,530 | 1,762,614 | 21 |
| 6 | AS | 3 | 28,192,395 | 27,977,462 | 1,799,226 | 33 |
| 7 | AS | 4 | 42,857,617 | 42,527,992 | 2,888,706 | 45 |
| 8 | AS | 5 | 25,662,109 | 25,442,846 | 2,265,516 | 36 |
| 9 | MG | 1 | 27,130,483 | 26,913,968 | 1,101,788 | 24 |
| 10 | MG | 2 | 22,722,363 | 22,525,660 | 1,055,808 | 51 |
| 11 | MG | 3 | 22,200,975 | 22,030,859 | 1,218,902 | 42 |
| 12 | MG | 4 | 28,386,955 | 28,137,734 | 2,326,586 | 38 |
| 13 | MG | 5 | 24,145,968 | 23,930,517 | 1,725,756 | 30 |
| **Total** | | | **355,917,017** | **352,872,111** | **22,749,072** | **99** |

***Figure S1:*** *Correlation plot to identify highly correlated predictors (>0.7) in each GLM (Evenness-AS; Evenness-MG; Richness-AS; Richness-MG). Numbers in brackets indicate the time-lag for each predictor in weeks. Hum = humidity; Temp = temperature.*

******

***Table S3:*** *Summary of minimal set of GLMs (excluding highly correlated predictors) evaluated per species and diversity metric. Subscripts in the GLMs indicate the time lag of the variable in weeks. AS = wood mice; MG = bank voles.*

| **Host species** | **Virus diversity metric** | **Model** |
| --- | --- | --- |
| AS | Evenness | 1. Temperature + humidity + host density 2. Temperature + rain + host density 3. Temperature + humidity 4. Temperature + host density 5. Temperature + rain 6. Rain + host density 7. Host density + humidity |
| MG | Evenness | 1. Temperature + humidity + rain 2. Temperature + humidity 3. Temperature + rain 4. Rain + host density 5. Rain + humidity |
| AS | Richness | 1. Temperature + rain 2. Temperature + host density 3. Humidity + host density 4. Rain + humidity |
| MG | Richness | 1. Temperature + host density + rain 2. Temperature + host density 3. Temperature + rain 4. Humidity + rain 5. Rain + host density |

***Table S4:*** *Viral read abundance of bacteriophage virus families (in descending order by viral abundance) detected in Wytham rodents. AF = Yellow-necked mice; AS = wood mice; MG = bank vole*

| **Virus family** | **Genome type** | **Host species** | **Viral read counts** |
| --- | --- | --- | --- |
| Leviviridae | ss+RNA | AS | 12356782 |
| Leviviridae | ss+RNA | MG | 6788793 |
| Leviviridae | ss+RNA | AF | 4409631 |
| Microviridae | ssDNA | AS | 1847068 |
| Microviridae | ssDNA | MG | 1560193 |
| Microviridae | ssDNA | AF | 345616 |
| Myoviridae | dsDNA | MG | 36388 |
| Myoviridae | dsDNA | AS | 22993 |
| Myoviridae | dsDNA | AF | 16253 |
| Siphoviridae | dsDNA | AF | 2197 |
| Podoviridae | dsDNA | AF | 544 |
| Podoviridae | dsDNA | AS | 488 |
| Siphoviridae | dsDNA | AS | 473 |
| Inoviridae | ssDNA | AF | 458 |
| Podoviridae | dsDNA | MG | 427 |
| Herelleviridae | dsDNA | AF | 316 |
| Siphoviridae | dsDNA | MG | 305 |
| Herelleviridae | dsDNA | MG | 144 |
| Inoviridae | ssDNA | AS | 56 |
| Inoviridae | ssDNA | MG | 34 |
| Herelleviridae | dsDNA | AS | 11 |

***Figure S2:*** *Rarefaction curves for all viruses, vertebrate-associated viruses, and bacteriophage.*

***Figure S3:*** *Host population density of the Wytham rodents between November 2016 and January 2017 as measured by minimum number known alive (MNKA) per hectare (ha). AF = Yellow-necked mice; AS = wood mice; MG = bank vole*

******

***Table S5:*** *Summary output from hierarchical PERMANOVA analysis. Viral community matrix was filtered to exclude very low and highly abundant viruses. Interval = Sampling interval; Species = Host species (AF, AS, or MG)*

| **All viruses** | | | | | | |
| --- | --- | --- | --- | --- | --- | --- |
|  | **Df** | **SumOfSqs** | **MeanSqs** | **F.model** | **R^2^** | **Pr(>F)** |
| Interval | 1 | 0.563 | 0.563 | 3.242 | 0.179 | 0.007 |
| Species | 2 | 0.895 | 0.448 | 2.575 | 0.284 | 0.043 |
| Interval:Species | 2 | 0.479 | 0.240 | 1.380 | 0.152 | 0.367 |
| Residuals | 7 | 1.217 | 0.174 |  | 0.386 |  |
|  |  |  |  |  |  |  |
| **Vertebrate-associated** | | | | | | |
|  | Df | SumOfSqs | MeanSqs | F.model | R^2^ | Pr(>F) |
| Interval | 1 | 0.365 | 0.365 | 2.399 | 0.122 | 0.050 |
| Species | 2 | 1.101 | 0.551 | 3.632 | 0.368 | 0.160 |
| Interval:Species | 2 | 0.466 | 0.233 | 1.533 | 0.155 | 0.445 |
| Residuals | 7 | 1.064 | 0.152 |  | 0.355 |  |
|  |  |  |  |  |  |  |
| **Bacteriophage** | | | | | | |
|  | Df | SumOfSqs | MeanSqs | F.model | R^2^ | Pr(>F) |
| Interval | 1 | 0.383 | 0.383 | 2.488 | 0.153 | 0.073 |
| Species | 2 | 0.604 | 0.302 | 1.961 | 0.241 | 0.262 |
| Interval:Species | 2 | 0.440 | 0.220 | 1.430 | 0.176 | 0.505 |
| Residuals | 7 | 2.504 | 0.154 |  | 0.430 |  |
|  |  |  |  |  |  |  |

***Figure S4:*** *Correlation coefficients for the top virus genera separating sampling intervals. A) Top 20 virus genera show for all viruses. Top 10 virus genera shown for B) Vertebrate-associated viruses and C) Bacteriophage.*

******

***Table S6:*** *Read abundance of virus genera from vertebrate-associated and bacteriophage virus families by host species and sampling interval.*

| Virus genera | Genome type | Species | Interval | Read abundance |
| --- | --- | --- | --- | --- |
| Alcyoneusvirus | dsDNA | AF | 3 | 356 |
| Alcyoneusvirus | dsDNA | AS | 4 | 2 |
| Alcyoneusvirus | dsDNA | MG | 2 | 2 |
| Alcyoneusvirus | dsDNA | MG | 3 | 2 |
| Allolevivirus | ss+RNA | AF | 3 | 14 |
| Allolevivirus | ss+RNA | AS | 3 | 15 |
| Allolevivirus | ss+RNA | MG | 4 | 152 |
| Bastillevirinae | dsDNA | AF | 3 | 11 |
| Bastillevirinae | dsDNA | MG | 2 | 5 |
| Bastillevirinae | dsDNA | MG | 3 | 74 |
| Betacoronavirus | ss+RNA | AF | 4 | 179 |
| Betaretrovirus | reverse transcribing | AF | 3 | 12 |
| Brockvirinae | dsDNA | AF | 3 | 28 |
| Brockvirinae | dsDNA | AS | 1 | 6 |
| Brockvirinae | dsDNA | MG | 3 | 6 |
| Brockvirinae | dsDNA | MG | 4 | 3 |
| Cardiovirus | ss+RNA | AF | 3 | 141 |
| Cardiovirus | ss+RNA | AF | 4 | 53 |
| Cardiovirus | ss+RNA | AF | 5 | 38 |
| Cardiovirus | ss+RNA | AS | 1 | 7 |
| Cardiovirus | ss+RNA | AS | 3 | 7343 |
| Cardiovirus | ss+RNA | AS | 4 | 35307 |
| Cardiovirus | ss+RNA | AS | 5 | 1 |
| Cardiovirus | ss+RNA | MG | 1 | 113 |
| Cardiovirus | ss+RNA | MG | 2 | 105 |
| Cardiovirus | ss+RNA | MG | 3 | 99 |
| Cardiovirus | ss+RNA | MG | 4 | 138 |
| Cardiovirus | ss+RNA | MG | 5 | 77 |
| Delepquintavirus | dsDNA | AF | 3 | 65 |
| Delepquintavirus | dsDNA | AF | 4 | 4 |
| Enterovirus | ss+RNA | AF | 3 | 2 |
| Enterovirus | ss+RNA | AS | 2 | 5 |
| Enterovirus | ss+RNA | MG | 4 | 4 |
| Eucampyvirinae | dsDNA | AF | 3 | 907 |
| Eucampyvirinae | dsDNA | AF | 4 | 2002 |
| Eucampyvirinae | dsDNA | AF | 5 | 12466 |
| Eucampyvirinae | dsDNA | AS | 1 | 4009 |
| Eucampyvirinae | dsDNA | AS | 2 | 3047 |
| Eucampyvirinae | dsDNA | AS | 3 | 1072 |
| Eucampyvirinae | dsDNA | AS | 4 | 10468 |
| Eucampyvirinae | dsDNA | AS | 5 | 4313 |
| Eucampyvirinae | dsDNA | MG | 1 | 3599 |
| Eucampyvirinae | dsDNA | MG | 2 | 3780 |
| Eucampyvirinae | dsDNA | MG | 3 | 2374 |
| Eucampyvirinae | dsDNA | MG | 4 | 16841 |
| Eucampyvirinae | dsDNA | MG | 5 | 9198 |
| Gokushovirinae | ssDNA | AF | 3 | 343 |
| Gokushovirinae | ssDNA | AF | 4 | 57 |
| Gokushovirinae | ssDNA | AF | 5 | 2 |
| Gokushovirinae | ssDNA | AS | 4 | 49 |
| Gokushovirinae | ssDNA | MG | 2 | 172 |
| Gokushovirinae | ssDNA | MG | 3 | 26 |
| Hunnivirus | ss+RNA | AF | 3 | 8 |
| Hunnivirus | ss+RNA | AF | 4 | 40 |
| Hunnivirus | ss+RNA | AF | 5 | 2 |
| Hunnivirus | ss+RNA | AS | 1 | 69 |
| Hunnivirus | ss+RNA | AS | 2 | 71 |
| Hunnivirus | ss+RNA | AS | 3 | 2 |
| Hunnivirus | ss+RNA | MG | 2 | 535 |
| Hunnivirus | ss+RNA | MG | 3 | 2734 |
| Hunnivirus | ss+RNA | MG | 4 | 5109 |
| Kunsagivirus | ss+RNA | AS | 2 | 61 |
| Kunsagivirus | ss+RNA | AS | 3 | 270 |
| Kunsagivirus | ss+RNA | AS | 4 | 692 |
| Kunsagivirus | ss+RNA | AS | 5 | 163 |
| Levivirus | ss+RNA | AS | 1 | 10 |
| Levivirus | ss+RNA | MG | 2 | 445 |
| Levivirus | ss+RNA | MG | 3 | 1382 |
| Levivirus | ss+RNA | MG | 4 | 205 |
| Levivirus | ss+RNA | MG | 5 | 2755 |
| Mamastrovirus | ss+RNA | AF | 3 | 74 |
| Mamastrovirus | ss+RNA | AF | 4 | 256 |
| Mamastrovirus | ss+RNA | AF | 5 | 6180 |
| Mamastrovirus | ss+RNA | AS | 2 | 1848 |
| Mamastrovirus | ss+RNA | AS | 5 | 24341 |
| Mamastrovirus | ss+RNA | MG | 1 | 42 |
| Mamastrovirus | ss+RNA | MG | 2 | 12 |
| Mamastrovirus | ss+RNA | MG | 4 | 849 |
| Mosavirus | ss+RNA | MG | 3 | 1602 |
| Picobirnavirus | dsRNA | AF | 3 | 2118401 |
| Picobirnavirus | dsRNA | AF | 4 | 1551531 |
| Picobirnavirus | dsRNA | AF | 5 | 2018628 |
| Picobirnavirus | dsRNA | AS | 1 | 3781449 |
| Picobirnavirus | dsRNA | AS | 2 | 2854309 |
| Picobirnavirus | dsRNA | AS | 3 | 2851733 |
| Picobirnavirus | dsRNA | AS | 4 | 3781218 |
| Picobirnavirus | dsRNA | AS | 5 | 2406864 |
| Picobirnavirus | dsRNA | MG | 1 | 1529028 |
| Picobirnavirus | dsRNA | MG | 2 | 1177104 |
| Picobirnavirus | dsRNA | MG | 3 | 2009804 |
| Picobirnavirus | dsRNA | MG | 4 | 2281786 |
| Picobirnavirus | dsRNA | MG | 5 | 2301954 |
| Picovirinae | dsDNA | AF | 3 | 289 |
| Picovirinae | dsDNA | AF | 4 | 2 |
| Sapelovirus | ss+RNA | MG | 1 | 20 |
| Sapelovirus | ss+RNA | MG | 2 | 6 |
| Sapelovirus | ss+RNA | MG | 4 | 3 |
| Sedoreovirinae | dsRNA | AS | 5 | 44 |
| Sedoreovirinae | dsRNA | MG | 3 | 5951 |
| Spounavirinae | dsDNA | AF | 3 | 216 |
| Spounavirinae | dsDNA | AF | 4 | 4 |
| Spounavirinae | dsDNA | AF | 5 | 9 |
| Spounavirinae | dsDNA | AS | 1 | 1 |
| Spounavirinae | dsDNA | AS | 5 | 2 |
| Spounavirinae | dsDNA | MG | 2 | 8 |
| Spounavirinae | dsDNA | MG | 5 | 6 |
| Tegunavirus | dsDNA | AF | 3 | 44 |
| Tevenvirinae | dsDNA | AF | 3 | 141 |
| Tevenvirinae | dsDNA | AF | 4 | 8 |
| Tevenvirinae | dsDNA | AF | 5 | 23 |
| Tevenvirinae | dsDNA | AS | 2 | 2 |
| Tevenvirinae | dsDNA | AS | 4 | 23 |
| Tevenvirinae | dsDNA | AS | 5 | 6 |
| Tevenvirinae | dsDNA | MG | 2 | 24 |
| Torovirus | ss+RNA | MG | 3 | 3744 |
| Torovirus | ss+RNA | MG | 4 | 91 |
| Twortvirinae | dsDNA | AF | 3 | 42 |
| Twortvirinae | dsDNA | AF | 4 | 6 |
| Twortvirinae | dsDNA | AS | 4 | 2 |
| Twortvirinae | dsDNA | MG | 1 | 6 |
| Twortvirinae | dsDNA | MG | 2 | 10 |
| Twortvirinae | dsDNA | MG | 3 | 26 |
| unclassified Astroviridae | ss+RNA | MG | 1 | 37 |
| unclassified Astroviridae | ss+RNA | MG | 3 | 87 |
| unclassified Astroviridae | ss+RNA | MG | 4 | 845 |
| unclassified Astroviridae | ss+RNA | MG | 5 | 41 |
| unclassified Hepeviridae | ss+RNA | AF | 5 | 4 |
| unclassified Hepeviridae | ss+RNA | AS | 4 | 12 |
| unclassified Hepeviridae | ss+RNA | AS | 5 | 984 |
| unclassified Hepeviridae | ss+RNA | MG | 1 | 4 |
| unclassified Hepeviridae | ss+RNA | MG | 2 | 223 |
| unclassified Hepeviridae | ss+RNA | MG | 3 | 214 |
| unclassified Hepeviridae | ss+RNA | MG | 4 | 778 |
| unclassified Hepeviridae | ss+RNA | MG | 5 | 2 |
| unclassified Inoviridae | ssDNA | AF | 3 | 381 |
| unclassified Inoviridae | ssDNA | AF | 4 | 59 |
| unclassified Inoviridae | ssDNA | AF | 5 | 18 |
| unclassified Inoviridae | ssDNA | AS | 4 | 56 |
| unclassified Inoviridae | ssDNA | MG | 2 | 34 |
| unclassified Leviviridae | ss+RNA | AF | 3 | 1761164 |
| unclassified Leviviridae | ss+RNA | AF | 4 | 1054730 |
| unclassified Leviviridae | ss+RNA | AF | 5 | 1593723 |
| unclassified Leviviridae | ss+RNA | AS | 1 | 2804896 |
| unclassified Leviviridae | ss+RNA | AS | 2 | 2047395 |
| unclassified Leviviridae | ss+RNA | AS | 3 | 1663540 |
| unclassified Leviviridae | ss+RNA | AS | 4 | 3020851 |
| unclassified Leviviridae | ss+RNA | AS | 5 | 2820075 |
| unclassified Leviviridae | ss+RNA | MG | 1 | 638105 |
| unclassified Leviviridae | ss+RNA | MG | 2 | 885087 |
| unclassified Leviviridae | ss+RNA | MG | 3 | 1132047 |
| unclassified Leviviridae | ss+RNA | MG | 4 | 2508658 |
| unclassified Leviviridae | ss+RNA | MG | 5 | 1619957 |
| unclassified Microviridae | ssDNA | AF | 3 | 133060 |
| unclassified Microviridae | ssDNA | AF | 4 | 55244 |
| unclassified Microviridae | ssDNA | AF | 5 | 156910 |
| unclassified Microviridae | ssDNA | AS | 1 | 487310 |
| unclassified Microviridae | ssDNA | AS | 2 | 376727 |
| unclassified Microviridae | ssDNA | AS | 3 | 383398 |
| unclassified Microviridae | ssDNA | AS | 4 | 298126 |
| unclassified Microviridae | ssDNA | AS | 5 | 301458 |
| unclassified Microviridae | ssDNA | MG | 1 | 246215 |
| unclassified Microviridae | ssDNA | MG | 2 | 196001 |
| unclassified Microviridae | ssDNA | MG | 3 | 311536 |
| unclassified Microviridae | ssDNA | MG | 4 | 394838 |
| unclassified Microviridae | ssDNA | MG | 5 | 411405 |
| unclassified Myoviridae | dsDNA | AF | 3 | 274 |
| unclassified Myoviridae | dsDNA | AF | 5 | 10 |
| unclassified Myoviridae | dsDNA | AS | 3 | 6 |
| unclassified Myoviridae | dsDNA | AS | 4 | 40 |
| unclassified Myoviridae | dsDNA | MG | 1 | 2 |
| unclassified Myoviridae | dsDNA | MG | 2 | 311 |
| unclassified Myoviridae | dsDNA | MG | 3 | 29 |
| unclassified Myoviridae | dsDNA | MG | 4 | 26 |
| unclassified Myoviridae | dsDNA | MG | 5 | 6 |
| unclassified Paramyxoviridae | ss-RNA | MG | 4 | 9 |
| unclassified Paramyxoviridae | ss-RNA | MG | 5 | 4 |
| unclassified Picobirnaviridae | dsRNA | AF | 3 | 1708 |
| unclassified Picobirnaviridae | dsRNA | AF | 4 | 2016 |
| unclassified Picobirnaviridae | dsRNA | AF | 5 | 8919 |
| unclassified Picobirnaviridae | dsRNA | AS | 1 | 2948 |
| unclassified Picobirnaviridae | dsRNA | AS | 2 | 3165 |
| unclassified Picobirnaviridae | dsRNA | AS | 3 | 5156 |
| unclassified Picobirnaviridae | dsRNA | AS | 4 | 4665 |
| unclassified Picobirnaviridae | dsRNA | AS | 5 | 3473 |
| unclassified Picobirnaviridae | dsRNA | MG | 1 | 8219 |
| unclassified Picobirnaviridae | dsRNA | MG | 2 | 8103 |
| unclassified Picobirnaviridae | dsRNA | MG | 3 | 12134 |
| unclassified Picobirnaviridae | dsRNA | MG | 4 | 10229 |
| unclassified Picobirnaviridae | dsRNA | MG | 5 | 5605 |
| unclassified Picornaviridae | ss+RNA | MG | 1 | 10 |
| unclassified Picornaviridae | ss+RNA | MG | 2 | 2 |
| Sapelovirus | ss+RNA | AF | 3 | 389 |
| Sapelovirus | ss+RNA | AS | 2 | 10 |
| Unclassified picornavirus | ss+RNA | AF | 3 | 2 |
| Unclassified picornavirus | ss+RNA | AF | 4 | 1170 |
| Unclassified picornavirus | ss+RNA | AS | 3 | 497 |
| Unclassified picornavirus | ss+RNA | AS | 4 | 985 |
| Unclassified picornavirus | ss+RNA | AS | 5 | 14 |
| Unclassified picornavirus | ss+RNA | MG | 2 | 2 |
| Unclassified picornavirus | ss+RNA | MG | 3 | 7 |
| Unclassified picornavirus | ss+RNA | MG | 4 | 2 |
| unclassified Podoviridae | dsDNA | AF | 3 | 196 |
| unclassified Podoviridae | dsDNA | AF | 4 | 52 |
| unclassified Podoviridae | dsDNA | AF | 5 | 5 |
| unclassified Podoviridae | dsDNA | AS | 1 | 6 |
| unclassified Podoviridae | dsDNA | AS | 2 | 344 |
| unclassified Podoviridae | dsDNA | AS | 4 | 73 |
| unclassified Podoviridae | dsDNA | AS | 5 | 65 |
| unclassified Podoviridae | dsDNA | MG | 1 | 10 |
| unclassified Podoviridae | dsDNA | MG | 2 | 18 |
| unclassified Podoviridae | dsDNA | MG | 3 | 38 |
| unclassified Podoviridae | dsDNA | MG | 4 | 26 |
| unclassified Podoviridae | dsDNA | MG | 5 | 335 |
| unclassified Reoviridae | dsRNA | AS | 2 | 2 |
| unclassified Reoviridae | dsRNA | AS | 3 | 51 |
| unclassified Siphoviridae | dsDNA | AF | 3 | 1852 |
| unclassified Siphoviridae | dsDNA | AF | 4 | 85 |
| unclassified Siphoviridae | dsDNA | AF | 5 | 134 |
| unclassified Siphoviridae | dsDNA | AS | 1 | 30 |
| unclassified Siphoviridae | dsDNA | AS | 2 | 23 |
| unclassified Siphoviridae | dsDNA | AS | 3 | 6 |
| unclassified Siphoviridae | dsDNA | AS | 4 | 322 |
| unclassified Siphoviridae | dsDNA | AS | 5 | 83 |
| unclassified Siphoviridae | dsDNA | MG | 1 | 1 |
| unclassified Siphoviridae | dsDNA | MG | 2 | 219 |
| unclassified Siphoviridae | dsDNA | MG | 3 | 9 |
| unclassified Siphoviridae | dsDNA | MG | 4 | 2 |
| unclassified Siphoviridae | dsDNA | MG | 5 | 37 |
| Vequintavirinae | dsDNA | MG | 1 | 63 |
| Vequintavirinae | dsDNA | MG | 2 | 28 |
| Vequintavirinae | dsDNA | MG | 3 | 11 |
| Vequintavirinae | dsDNA | MG | 5 | 12 |

***Table S7:*** *Number of assembled virus contigs that are similar in length to complete virus genomes in the most abundant virus families in observed Wytham rodent virome.*

| **Group** | **Virus family** | **Number of assembled**  **contigs (length)** |
| --- | --- | --- |
| Vertebrate-associated | *Picobirnaviridae* (dsRNA) | 1) 114 (2100-3000bp^a^)  2) 154 (1200-1900bp^b^) |
| Vertebrate-associated | *Picornaviridae* (ss+RNA) | 8 (>4500bp) |
| Bacteriophage | *Leviviridae* (ss+RNA) | 21 (>3500bp) |
| Bacteriophage | *Microviridae* (ssDNA) | 9 (>4000bp) |

^a^ range based on segment 1

^b^ range based on segment 2

***Table S8:*** *Description of Picornavirus genomes assembled in Wytham rodents. Contigs labelled “a” and “b” correspond to distinct genome sequences belonging to the same genera.*

| **Contig name** | **Genus** | **Genome length** | **Complete**  **(Y/N)** |
| --- | --- | --- | --- |
| Wytham cardiovirus | *Cardiovirus* | 8205 bp | Y |
| Wytham mosavirus | *Mosavirus* | 8114 bp | Y |
| Wytham kunsagivirus | *Kunsagivirus A* | 4851 bp | N |
| Wytham hunnivirus (a) | *Hunnivirus* | 7737 bp | Y |
| Wytham hunnivirus (b) | *Hunnivirus* | 7405 bp | Y |
| Wytham sapelovirus | *Sapelovirus* | 7553 bp | Y |
| Unclassified picornavirus (a) | Unclassified picornavirus | 9085 bp | Y |
| Unclassified picornavirus (b) | Unclassified picornavirus | 9217 bp | Y |

***Figure S5:*** *Picornavirus species abundance, evenness, and richness based on eight picornavirus genomes assembled in this study. AS = wood mice (green), AF = yellow-necked mice (blue), MG = bank voles (orange)*

******

***Figure S6:*** *Variables used to explore predictors of picornavirus diversity. A) Viral evenness and richness of picornaviruses, together with host population density (measured as MNKA per ha) for wood mice (AS) and bank voles (MG). Open circles indicate imputed values, while filled circles correspond to observed values. B) Daily inferred temperature (mean), humidity (mean), and rainfall (summed per 24 hour period) in Wytham woods using two microclimate stations.*

***
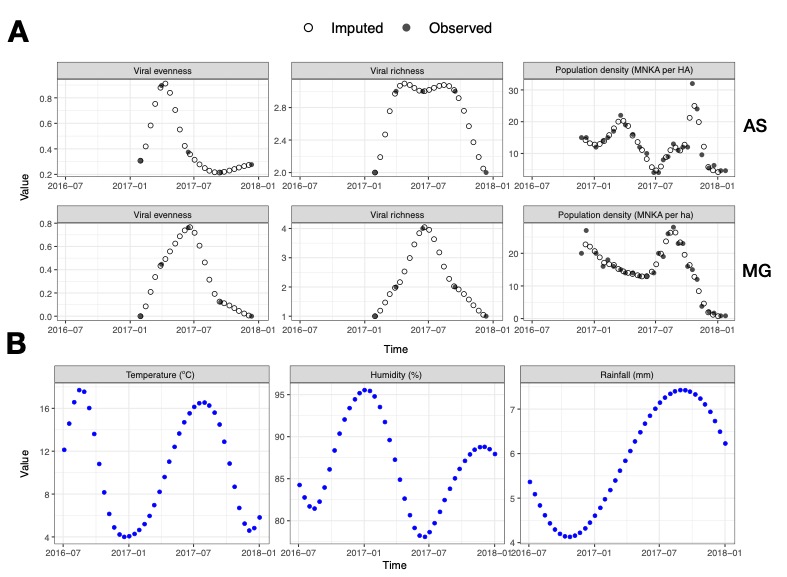
***

***Table S9:*** *Summary of cross-correlation analysis of four key variables with viral evenness or richness. Lags (in weeks) were identified based on the maximum significant residual auto-correlation values (r_xy_).*

|  | **AS** | | **MG** | |
| --- | --- | --- | --- | --- |
| **Variables** | Evenness  (Lag in weeks; r_xy_) | Richness  (Lag in weeks; r_xy_) | Evenness  (Lag in weeks; r_xy_) | Richness  (Lag in weeks; r_xy_) |
| Temperature | 14; 0.64 | 0; 0.77 | 4; 0.75 | 2; 0.80 |
| Humidity | 10; 0.63 | 0; -0.76 | 0; -0.77 | 0; -0.88 |
| Rain | 0; -0.39 | 8; 0.39 | 14; -0.40 | 10; 0.38 |
| Host density | 12; -0.42 | 0; -0.20 | 8; -0.72 | 0; -0.61 |

***Table S10:*** *Scaled coefficients of the best fit models.* Subscripts next to predictor variables indicate species and/or time lags (in weeks). *Predictors are mean-centered and scaled by one standard deviation. Significance levels indicated by *** p < 0.001; * p < 0.05*

| **Predictor** | **Evenness (AS)** | **Evenness (MG)** | **Richness (AS)** | **Richness (MG)** |
| --- | --- | --- | --- | --- |
| Temperature_14_ | -1.24***  [-1.36, -1.13] |  |  |  |
| Host density_12 (AS)_ | -0.72***  [-0.83, -0.61] |  |  |  |
| Rain_14_ |  | -0.39 ***  [-0.49, -0.29] |  |  |
| Humidity |  | -1.04***  [-1.14, -0.93] |  |  |
| Temperature_4_ |  | -0.22 **  [-0.35, -0.08] |  |  |
| Temperature |  |  | 0.96 ***  [0.86, 1.07] |  |
| Host density_0, AS_ |  |  | 0.48 ***  [0.37, 0.58] |  |
| Temperature_2_ |  |  |  | 0.40 *** [0.32, 0.48] |
| Host density_0,MG_ |  |  |  | 0.75 *** [0.67, 0.82] |
